# Activation of Early Proinflammatory Responses by TBEV NS1 Varies between the Strains of Various Subtypes

**DOI:** 10.3390/ijms24021011

**Published:** 2023-01-05

**Authors:** Elizaveta Starodubova, Ksenia Tuchynskaya, Yulia Kuzmenko, Anastasia Latanova, Vera Tutyaeva, Vadim Karpov, Galina Karganova

**Affiliations:** 1 Engelhardt Institute of Molecular Biology, Russian Academy of Sciences, 119991 Moscow, Russia; 2 FSASI “Chumakov FSC R&D IBP RAS” (Institute of Poliomyelitis), prem. 8, k.17, pos. Institut Poliomyelita, poselenie Moskovskiy, 108811 Moscow, Russia

**Keywords:** tick-borne encephalitis virus (TBEV), nonstructural protein 1 (NS1), pro-inflammatory cytokines

## Abstract

Tick-borne encephalitis (TBE) is an emerging zoonosis that may cause long-term neurological sequelae or even death. Thus, there is a growing interest in understanding the factors of TBE pathogenesis. Viral genetic determinants may greatly affect the severity and consequences of TBE. In this study, nonstructural protein 1 (NS1) of the tick-borne encephalitis virus (TBEV) was tested as such a determinant. NS1s of three strains with similar neuroinvasiveness belonging to the European, Siberian and Far-Eastern subtypes of TBEV were studied. Transfection of mouse cells with plasmids encoding NS1 of the three TBEV subtypes led to different levels of NS1 protein accumulation in and secretion from the cells. NS1s of TBEV were able to trigger cytokine production either in isolated mouse splenocytes or in mice after delivery of NS1 encoding plasmids. The profile and dynamics of TNF-α, IL-6, IL-10 and IFN-γ differed between the strains. These results demonstrated the involvement of TBEV NS1 in triggering an immune response and indicated the diversity of NS1 as one of the genetic factors of TBEV pathogenicity.

## 1. Introduction

Tick-borne encephalitis virus (TBEV) belongs to *Flaviviridae* family of viruses that cause serious human disease. Infection with TBEV can lead to different clinical manifestations and neurological sequelae. The course, the severity and complications of tick-borne encephalitis (TBE) depend on a combination of multiple factors that include viral dose, subtype/strain, as well as the host peculiarities. Three major subtypes of TBEV have been identified: European, Siberian, Far-Eastern and the recently isolated Baikalian and Himalayan subtypes [1,2,3,4]. In general, viruses of the European subtype cause rather light clinical manifestations with a mortality rate of less than 2% and patients with the Siberian subtype have generally mild forms of disease, but chronic forms of disease have also been registered, whereas infections with the Far-Eastern subtype cause the most severe manifestations of TBE and the highest mortality [5,6]. Clinical manifestations of TBE are well characterized, but the systemic analysis of disease severity factors are limited [7,8,9,10]. Therefore, an estimation of the factors defining the severity of TBE is vital. Analysis of viral genomes have revealed that specific mutations in viral proteins may have an impact on the TBEV pathogenesis [11,12,13].

The TBEV genome encodes one polyprotein that is processed into three structural proteins (C, M and E) and seven non-structural (NS) proteins (NS1, NS2A, NS2B, NS3, NS4A, NS4B and NS5) by cellular and viral proteases [14]. The specific roles of each protein in the pathogenicity of virus are still completely unknown [13]. The importance of structural proteins for non-viremic transmission between ticks [15,16,17] has been shown, whereas non-structural proteins were involved in cytotoxicity [18]. It has been shown in many studies that the determinants of viral neuropathogenesis were located in the E protein [12,13]. In addition, mutations in the NS5 protein have been shown to contribute to the attenuation of virulence and host–cell interactions [13,19,20]. Amino acid substitutions in NS3 are known to affect the budding of viral particles, thus leading to diminished pathogenicity [11,21]. The data on other NS proteins of TBEV in virus–host interactions are very scarce. 

Among the non-structural proteins, NS1 attracts attention by its unusualness. NS1 protein is a 46 kDa glycoprotein that is initially synthetized as a monomer, which forms a dimer in ER and is transferred to the cell surface where it forms hexamers, which are secreted [22]. NS1 is highly conserved protein among flaviviruses; therefore, NS1s of different viruses of the family have similar structure and functions [23]. Flaviviral intracellular NS1 protein is involved in the viral replication process and virus assembly, whereas the secreted hexamers trigger various effects in the host immune system such as immune evasion [24,25] as well as an induction of the immune response [24]. It should be noted that the majority of data about NS1 properties were obtained on dengue and other mosquito-borne flaviviruses, whereas studies of TBEV NS1 are very limited. For dengue infection, the detection of secreted NS1 in serum has been used as an early diagnostic marker, and NS1’s involvement in vascular leakage, coagulopathy and thrombocytopenia have been shown [26]. Several cytokines, including TNF-α, IL-6, IL-10 and IFN-γ, have been proposed as potential predictors of dengue severity [23]. Dengue virus NS1 is able to stimulate production of TNF-α and IL-10 in human primary macrophages [27], and its intravenous injection resulted in elevated levels of TNF-α and IL-6 in mice serum [28]. In TBE patients, elevated levels of IFN-γ, TNF-α and IL-6 have been detected and are associated with disease severity [10,29,30,31]. Low levels of IL-10 in the cerebrospinal fluid correlated with more severe encephalitis [32]. TBEV NS1 were shown to be immunogenic [33] and able to induce oxidative stress [34] and expression of immunoproteasome subunits [35]; however, no data on triggering the cytokines exist.

In the present study, we focused on revealing the role of TBEV NS1 protein in triggering TNF-α, IL-10, IL-6 and IFN-γ production. NS1s of three TBEV strains belonging to the European, Siberian and Far-Eastern subtypes are included in the study. Since disease severity is associated with TBEV subtype only to a certain extent, and the pathogenic properties of individual strains belonging to one subtype varyies from low to high [11,36,37], strains with a similar neuroinvasiveness index were used. The secreted form of NS1 was able to stimulate cytokine production in naïve immune mouse cells. Injection of the NS1-encoding plasmid led to activation of cytokine production in mice. The profile and dynamics of cytokine activation differed between the three subtypes of NS1 of strains. These results highlight the importance of studying NS1 for understanding the pathogenicity of TBEV.

## 2. Results

### 2.1. Expression of Recombinant NS1 Protein of TBEV Strains of Three Subtypes in Mammalian 4T1 Cell Lines 

**Figure 1:**
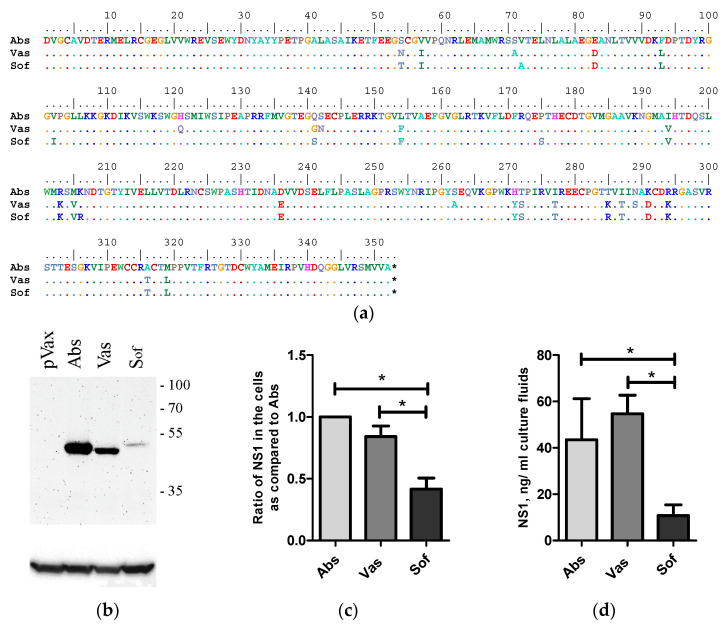
Alignment and expression of NS1 of strains of three TBEV subtypes in 4T1 cells. Alignment of NS1 of strains Absettarov (Abs) of the European subtype, Vasilchenko (Vas) of the Siberian subtype and Sofjin-Chumakov (Sof) of the Far-Eastern subtype (**a**). 4T1 cells were transfected with the plasmid-encoded NS1 of strains Absettarov (Abs) of the European subtype, Vasilchenko (Vas) of Siberian subtype and Sofjin-Chumakov (Sof) of the Far-Eastern subtype or pVax vector. After two days, the cells were lysed and analyzed by Western blot with anti-NS1 antibodies clone 4C4 (**b**); the positions of molecular mass markers are given on the right in kDa. The ratio of NS1 amount in cells normalized to actin as compared to Abs (data of more than 3 repeats were present) (**c**). The amount of NS1 secreted into cell culture fluids was measured by ELISA (**d**). Statistically significant differences (* *p* < 0.05) were derived by the Student’s *t*-test.

The NS1 genes were amplified from the viral genome RNA of TBEV strains Absettarov (Abs) of the European subtype, Vasilchenko (Vas) of the Siberian subtype and Sofjin-Chumakov (Sof) of the Far-Eastern subtype using reverse transcription and PCR with specific primers. Selected strains can cause lethal infection in mice [38,39]. In the present study, similar neuroinvasiveness indexes were demonstrated for the three stains: Abs: 0.5 ± 0.5, Vas: 0.67 ± 0.5 and Sof: 0.62 ± 0.5. Genes of NS1 were cloned into the pVax vector. The three encoded NS1 proteins differed in 28 amino acid positions (Figure 1a). 

Expression of NS1 protein was tested in 4T1-murine mammary carcinoma cell lines with epithelial morphology syngenic to BALB/c mice [40]. The 4T1 cells were transfected with NS1-encoding plasmids and the amount of NS1 protein in cell lysates was determined by Western blot analysis and in cell culture fluids by sandwich ELISA. NS1 proteins of all variants were detected in cells and in cell culture fluids. No statistical differences in the expression (Figure 1b,c) and secretion (Figure 1d) of Abs and Vas were observed. The amount of Sof NS1 protein was statistically lower compared to the amount of Abs and Vas NS1s both in the cell lysates (Figure 1b,c) and culture fluids of 4T1 cells 48 h after transfection (Figure 1d).

### 2.2. Secreted NS1 Produced in Mammalian Cells Can Activate Cytokine Production in Naive Mouse Splenocytes 

**Figure 2:**
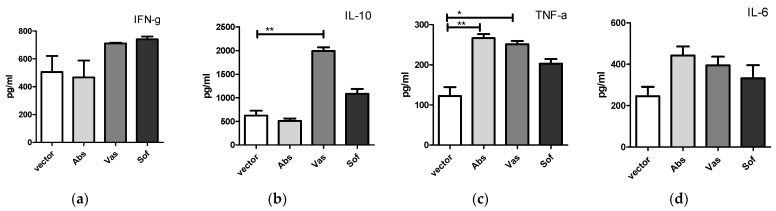
Cytokine secretion by naïve mouse splenocytes after incubation with NS1 proteins of Absettarov (Abs), Vasilchenko (Vas) and Sofjin-Chumakov (Sof) strains of three TBEV subtypes. Splenocytes were incubated with 1 μg/mL of NS1 for 20 h, and levels of IFN-γ (**a**), IL-10 (**b**), TNF-α (**c**) and IL-6 (**d**) in cell culture fluids were measured by ELISA. Each bar represents the mean value with SD of three mice samples run in duplicates. Statistically significant differences (* *p* < 0.05, ** *p* < 0.01) determined by the Student’s *t*-test of individual strains compared to the vector are labeled.

Next, we tested if the secreted form of NS1 can affect cytokine production by immune cells in vitro. For this, purified NS1 proteins and splenocytes of naïve mice were used. The NS1 proteins of the three strains were purified by FPLC from cell culture fluids of plasmid transfected HEK 293T cells (Supplementary Figure S1). Coomassie staining of NS1 samples resolved in SDS-PAGE demonstrated a high purity of the resulting probes (Supplementary Figure S1C). Mouse splenocytes were incubated with purified NS1 proteins, and TNF-α, IL-6, IFN-γ and IL-10 were measured in culture fluids using ELISA. After 20 h of incubation, the levels of IFN-γ and IL-6 in culture fluids of NS1 treated splenocytes did not differ from control treated samples. Secretion of TNF-α was detected after stimulation with Abs and Vas NS1 variants (Figure 2). IL-10 production by splenocytes was triggered only by Vas NS1.

### 2.3. Cytokine Production in Mice after Injection of Plasmids Encoding NS1 Differs between the Strains of Three TBEV Subtypes

**Figure 3:**
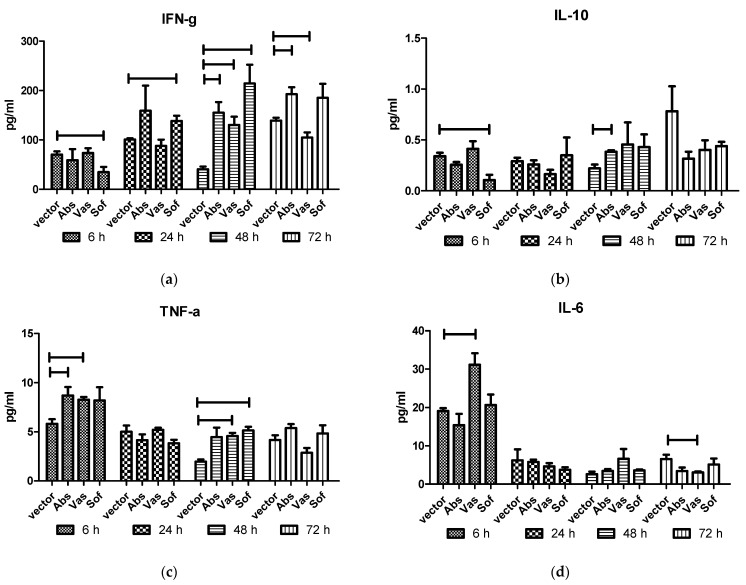
Cytokines in sera of mice after injection of NS1 genes of the strains of three TBEV subtypes. Plasmids encoding NS1 of Absettarov (Abs) of the European subtype, Vasilchenko (Vas) of the Siberian subtype and Sofjin-Chumakov (Sof) of the Far-Eastern subtype strains or pVax vector (vector) were intramuscularly injected into BALB/c mice, and levels of IFN-γ (**a**), IL-10 (**b**), TNF-α (**c**) and IL-6 (**d**) in sera were measured by ELISA. Each bar represents the mean value with SD of three pools of 4–5 mice sera that were run in duplicates. Statistically significant differences (*p* < 0.05) derived by the Student's *t*-test of individual strains compared to vector are labeled.

To assess the in vivo effect of NS1 protein on cytokine production, the plasmids encoding NS1 proteins of three TBEV strains or the empty vector were intramuscularly injected into BALB/c mice. Cytokines were measured in mice sera at 6, 24, 48 and 72 h after injection (Figure 3) by ELISA. In general, injection of all NS1 genes caused an increase in the levels of TNF-α at 6 h and 48 h, IFN-γ at 48 h, and a decrease in the level of IL-10 at 72 h compared to vector injected mice (Table 1). Individually, the NS1 of different TBEV strains displayed some dissimilarities between cytokine profile and dynamics. In Abs NS1-injected mice, increased IL-10 levels at 48 h and a longer persistence of IFN-γ levels at 48 h and 72 h were observed. In Vas NS1-injected mice, the IL-6 level increased after 6 h and decreased after 72 h, which was accompanied by an IFN-γ decrease at 72 h. The Sof NS1 differed, with decreased IFN-γ and IL-10 levels after 6 h, followed by earlier increase in IFN-γ levels after 24 h.

### 2.4. Complement Activation in Mice after NS1 Gene Injection Differs between NS1 of Strains of Three TBEV Subtypes

**Figure 4:**
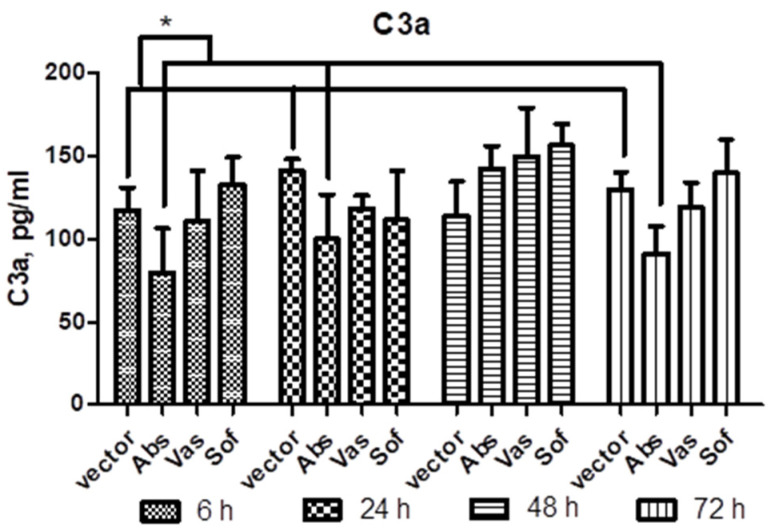
C3a in sera of mice after injection of NS1 genes of strains of three subtypes. Plasmids encoding NS1 of Absettarov (Abs), Vasilchenko (Vas) and Sofjin-Chumakov (Sof) strains or pVax vector (vector) were intramuscularly injected into BALB/c mice and levels of C3a in sera were measured by ELISA. Each bar represents the mean value with SD of three pools of 4–5 mice sera that were run in duplicate. Statistically significant differences (* *p* < 0.05) derived by the Student’s *t*-test of individual strains as compared to vector are labeled.

In order to examine the involvement of secreted NS1 in the innate response, the complement activation was tested. For this, sera of mice after injection of NS1 genes of TBEV strains Absettarov (Abs) of the European subtype, Vasilchenko (Vas) of the Siberian subtype and Sofjin-Chumakov (Sof) of the Far-Eastern subtype were tested for C3a component of complement by ELISA. Decreased C3a levels were observed only in Abs NS1 injected mice at 6 h, 24 h and 72 h (data pooled together) when compared to vector mice (Figure 4).

## 3. Discussion

Identification of factors determining the course and severity of TBE is of great importance. Among the major viral determinants of TBEV pathogenesis, the envelope protein E, nonstructural protein 5 and the 3′ untranslated region are noted [12]. A genome-wide evolutionary study revealed that adaptive changes in NS1 underlie epidemic and pathogenic diversities between TBEV subtypes [41]. There is no information about the involvement of TBEV NS1 in virus pathogenicity, but the NS1 protein of other flaviviruses is recognized as one of the pathogenic factors [23,25,26].

In our study, we tested NS1 of three strains of the European, Siberian and Far-Eastern subtypes, which have been shown to have the same neuroinvasiveness (0.5 lg) in mice. The three NS1 proteins differed in 28 amino acid positions. Sites of TBEV NS1 glycosylation [42] were unchanged. Two previously described mutations (T277V and E279G) in the dimerization domain of NS1 were suspected to be associated with the chronic form of disease [14]. These mutations were absent in the studied strains. In other analyses of TBEV strains with variable pathogenicity, a key substitution in NS1 S141G [11,43] has been revealed. Serine, the hydrophilic amino acid, was specific to highly virulent strains and glycine was specific to low virulent ones [44]. In the respective position in Abs, Vas and Sof, glutamine, glycine and serine were presented, respectively. Thus, the Vas strain can be attributed to low virulent strains and Sof to highly virulent ones. The Abs probably should be more similar to a highly virulent strain, since glutamine is an amino acid with a polar uncharged side chain, similar to serine. However, this contradicts data on the neuroinvasiveness of these strains in mice. Therefore, a further study of the substitutions at this position may reveal additional virulence determinants. In another study, the importance of dengue virus NS1 phosphorylation has been observed [45]. Some mutations significantly decreased the production of infectious virus but did not affect relative levels of intracellular NS1 expression or secretion. Only one mutation led to a significant reduction in detectable NS1 dimers in dengue virus-infected cells. In studied NS1 variants, there are 12 positions where the possible variations in phosphorylation of S, Y or T may occur. A total of six possible sites of phosphorylation are presented in Abs, five sites in Vas and ten in Sof. Thus, the differences in phosphorylation of NS1 in the studied strains could be one of the reasons for the different expression and secretion levels observed in this study.

Activation of innate and pro-inflammatory responses are the first host immune reactions to infection. Most of the studies of immune parameters have been assessed during the second phase of TBE [10,46,47]. Data on early immune responses to TBEV infection in human and mice experimental models are very limited [9,39]. In the present study, we focused on evaluation of the possible involvement of TBEV NS1 protein in triggering cytokines that were detected in TBE severe cases or were stimulated by NS1 of other flaviviruses. TNF-α, IL-6, IL-10 and IFN-γ were selected. These cytokines were upregulated in serum or cerebrospinal fluid of TBE patients. Immune correlations between TBE severity have been suggested only in a few studies. Inflammatory factors including TNF-α and IL-6 were associated with an unfavorable outcome of the disease [10,29,30]. High levels of IFN-γ and IL-6 in cerebrospinal fluid were suggested as one of the risk factors for incomplete recovery in TBE children [31]. As for IL-10, increased CSF levels were detected in TBE patients; however, these levels were lower in patients with more severe disease outcomes [32].

First, we tested the ability of secreted TBEV NS1 to stimulate cytokines in vitro in naïve mouse splenocytes. For dengue virus, it has been shown that the NS1 expressed in Drosophila S2 cells was able to stimulate production of IL-10 and TNF-α in human primary macrophages in complexes with human high-density lipoproteins, but not alone [27]. It should be noted that NS1 secreted from mammalian cells is a high density lipoprotein [48]; however, in some studies, authors failed to detect secretion of NS1 from insect cells [49]. In the present study, we used NS1-containing high-molecular weight fractions of cell culture fluids, collected from NS1 transfected human cells, where NS1 was represented in its natively secreted hexameric form; thus, it was potent in cytokine stimulation of naïve mouse splenocytes. As for dengue NS1, we also detected the production of TNF-α and IL-10 by mouse splenocytes. Immunophenotyping could be applied to reveal the cytokine secreting cells. Furthermore, in this study, we tested selected cytokines in mice serum shortly after NS1-encoding plasmid administration. TBEV initial infection and replication occurs in dendritic cells (DCs), macrophages and neutrophils [50]. NS1-encoding plasmid injected into mice transfects myocytes, keratinocytes, and DCs [51], and expression of encoded antigens could be detected in the site of injection already after 2 h and for some antigens this could last up to twenty days [52]. Therefore, we used DNA plasmid intramuscular injections for modeling the early stage of infection and tested individual NS1 effects. Changes in TNF-α, IL-6, IL-10 and IFN-γ in mice sera were observed after NS1-encoding plasmid injections.

The data obtained in this study on immune cells and in mice had both common trends and differences. The secretion of TNF-α was triggered in splenocytes and in mice for all NS1 variants. This points to the involvement of TBEV NS1 in inflammatory processes that are correlated with virus pathogenesis. In another published study performed on a mouse model, recombinant dengue NS1 produced in human embryonic kidney (HEK) 293 cells or in S2 cells was able to stimulate TNF-α and IL-6 three days after intravenous administration [28]. However, in our study, IL-6 production was induced only in mice 6 h after NS1-encoding plasmid injection. No IFN-γ secretion was observed in splenocytes and a gradual increase in IFN-γ was seen in mice. This likely demonstrates an evolving immune response against the foreign antigen, as it has been previously shown that TBEV NS1 is immunogenic after DNA immunization [33,53]. In mice, IL-10 levels were not significantly affected, but tended to decrease 72 h post-injection. Reduced levels of IL-10 in cerebrospinal fluid on days after the onset of encephalitis were observed in patients with encephalitis but not in patients with meningeal disease [32]. It is possible that the secreted NS1 could mediate this downregulation. However, at earlier time points, variation in IL-10 levels was observed for individual strains; it was reduced in mice for Sof at 6 h, elevated in splenocytes for Vas at 20 h and increased for Abs at 48 h. It should be noted that cytokine secretion is a process that can change rapidly over time. In another study, a comparison of two TBEV strains with similar virulence rates also showed a significant variation in the process of infection and immune response activation in mice [39]. Based on the results obtained in the present study, we can speculate TBEV NS1 involvement in triggering pro-inflammatory and adaptive immune responses, as shown by upregulated TNF-a and IFN-γ in all studied strains. However, the levels of IL-6 and IL-10 can underlie the differences between the strains or subtypes of TBEV. For other members of the flavivirus group, i.e., dengue virus, it has been suggested that the role of NS1 in pathogenesis is also strain-dependent [45].

As the part of the innate immune response, the activation of the complement system was tested. The interaction of TBEV with complement is unknown. However, in dengue infection, high levels of NS1, C3a, C5a and soluble C5b-9 present in the plasma of infected patients served as predictors of severe forms of disease [54,55]. In our study, analysis of the C3a amount in mice sera revealed decreased C3a levels in mice which had received plasmid encoding NS1 of the Absettarov strain compared to vector group. This result suggested that TBEV NS1 can affect the complement and a more detailed study of different complement factors with various viral strains is needed.

Summarizing the results of present study, we have revealed similarities and differences between studied NS1-driven features of strains of three TBEV subtypes. Expression and secretion of NS1 in the three strains were different. All studied TBEV NS1 proteins were involved in triggering the adaptive and pro-inflammatory immune responses. However, effects of NS1 of the three strains differ significantly for IL-6 and Il-10 either in the profile or in the dynamics. It should be mentioned that in the present study, only a single strain of each subtype was tested, and we cannot claim that the discovered properties are a characteristic of the subtype as a whole. To answer this question, more strains should be involved in the study. However, the presented results have demonstrated that TBEV NS1 is involved in cytokine triggering that varies between the strains. Thus, the variability in NS1 can be considered as an important genetic factor of TBEV pathogenicity.

## 4. Materials and Methods

### 4.1. Viruses

Three TBEV strains belonging to the three different TBEV subtypes were used in this study. The Absettarov strain (GenBank # KU885457) was isolated from the blood of an infected patient in the Leningrad region, Russia in 1951 (belonging to the European TBEV subtype), the Vasilchenko strain (GenBank # L40361) was also isolated from the blood of an infected patient in the Novosibirsk region, Russia in 1961 (belonging to the Siberian TBEV subtype) and the Sofjin-Chumakov strain (GenBank # KC806252) was isolated from the brain of a deceased TBE patient in the Primorsky Krai region in 1937 (belonging to the Far Eastern TBEV subtype).

The neuroinvasiveness index for female BALB/c mice (12–14 g) was estimated as the ratio between the number of infectious viral particles obtained by plaque assay in PEK cells (PFU) determined as described earlier [56], and the 50% lethal dose (LD50) of the virus after subcutaneous (s/c) inoculation was calculated according to the Kerber method [57].

### 4.2. Cloning of NS1 Genes and Plasmid Purification

Isolation, storage and propagation of the TBEV strains from the laboratory collection of the FSBSI (Chumakov FSC R&D IBP RAS) has been described previously [39,58,59]. Viral RNA was isolated using Trireagent LS (Sigma-Aldrich, Burlington, VT, USA) followed by chloroform extraction. For reverse transcription with SuperScript III (Invitrogen, Waltham, MA, USA), specific primers were used for the Absettarov and Vasilchenko strains (5′-atgtattcatctgttcgtcc-3′) and the Sofjin-Chumakov strain (5′-cagagcctgggtgcatgtcc-3′). Then, primers with restriction sites *BamHI* and *EcoRI* for forward and reverse primers, respectively, were used to amplify the NS1 genes. PCR fragments were cloned into the pVax vector. Sequences of the resulting plasmids were confirmed by sequencing. Plasmids were purified using Plasmid EndoFree Kits (Qiagen, Venlo, The Netherlands).

### 4.3. Cell Lines and Transfection

HEK293T and 4T1 (CRL-2539™, ATCC) cell lines were cultured in DMEM (Paneco, Russia) supplemented with 10% FBS (Hyclone Cytiva, Marlborough, MA, USA) and penicillin/streptomycin at 37 °C with a 5% CO_2_ humidified atmosphere. The day before transfection, cells were plated, and the next day were transfected using Lipofectamin LTX reagent (Thermo Fisher Scientific, Waltham, MA, USA) according to the manufacturer’s instructions.

### 4.4. Western Blot

Cell lysates were loaded onto 10% SDS-PAGE and then blotted on a nitrocellulose membrane. Blots were blocked in PBS with 0.01% Tween-20 and 5% dry milk and stained with anti-NS1 antibodies (clone 4C4 or 29F10, BioSan, Novosibirsk, Russia) or anti-actin (clone AC-15, Sigma) in blocking buffer followed by incubation with secondary HRP-conjugated anti-mouse antibodies (Jackson ImmunoResearch, Cambridge, UK). Blots were developed with ECL reagent (Amersham, Cytia). Signals were registered onto X-ray film (FujiFilm) or by ChemiDoc. Images were analyzed by ImageJ software (https://imagej.nih.gov/ij/).

### 4.5. NS1 Protein Purification

Protein was obtained as previously described in [35]. In brief, HEK293T cells were transfected with the plasmids encoding NS1 and grown for two days in serum-free medium. Then, cell culture fluids were collected, cell debris was removed by centrifugation and samples were concentrated by VivaSpin (Sartorius, Mw 100 kDa). Concentrated samples were subjected to an FPLC column of Sephadex 200. High-molecular weight protein fractions (90 kDa–300 kDa) were collected and analyzed by Western blot with anti-NS1 antibodies (clone 4C4, Biosan, Novosibirsk, Russia) (Supplementary Figure S1A,B). Fractions with maximum amounts of NS1 were pooled, concentrated with VivaSpin (Sartorius, Mw 5 kDa) and analyzed in gel followed by Coomassie blue staining. Cell culture fluids of cells transfected with pVax were processed the same as NS1-containing samples and were used as a control.

### 4.6. Animals and Treatment

Susceptible inbred mice of the strain BALB/c (State Institution Scientific Center of Biotechnology, branch “Stolbovaya”, Moscow, Russia) were used in this study. The animals were kept and treated in accordance with the international recommendations for the treatment of laboratory animals (CIOMS recommendations, 1985, the Directive 2010/63/EU, and Appendix A to the European Convention ETS No. 123). The bioethics committee of FSBSI Chumakov FSC R&D IBP RAS (protocol #17 from 1 September 2016) approved all experimental procedures performed on animals.

Mice splenocytes were purified as described previously [52]. In brief, spleens were homogenized and cells were filtered through a nylon filter, then red blood cells were lysed in ACK Lysis buffer. A concentration of 2.5 × 10^5^ of splenocytes were incubated with 1 mg/mL NS1 protein for 20 h in RPMI supplemented with 5% FBS.

Plasmid DNA was injected intramuscularly into two hind legs using an insulin syringe. Each mouse received two injections of 50 μg DNA in 50 μL of TE buffer.

### 4.7. ELISA

For NS1 measurement, sandwich ELISA was used. Coating anti-NS1 antibody clone 4C4 (Biosan, Novosibirsk, Russia) in carbonate buffer were sorbed onto a high-binding 96-well plate (Grainer). Blocking was performed in PBS with 0.05% Tween-20 and 5% BSA. Detection of NS1 was performed with anti-NS1 clone 29G9 (Biosan, Novosibirsk, Russia) conjugated to biotin followed by incubation with streptavidin-HRP conjugate. TMB (Abcam, Cambridge, UK) was use as a substrate.

Cytokines levels (IFN-γ, IL-10, TNF-α and IL-6) in mice sera were measured by ELISA kits (Invitrogen) and C3a in mice sera was measured by an ELISA kit (Cloud-Clone Corp., Wuhan, China) according to the manufacturer’s instructions. For tests, mice sera were pooled (4–5 mice) and run in duplicate.

### 4.8. Statistical Analysis and Software

All data were presented as mean with SD. Statistical differences were analyzed by one-way ANOVA in combination with post Tukey’s test (=0.05) to assess the difference between any two groups by the unpaired *t*-test by using GraphPad Prism 8.4 statistical software (GraphPad Software, Inc., San Diego, CA, USA). *p* < 0.05 and *p* < 0.01 were considered statistically significant.

## 5. Conclusions

The results of this study demonstrate that TBEV NS1 plays an important role, not only in the replication of the virus, but also in affecting the early cytokine production, and this differs between viral strains. These results have highlighted important aspects that could be studied further to reveal the NS1-related pathogenicity of TBEV. This includes the study of the genetic diversity of NS1 and its interaction with innate and inflammatory host responses.

## Figures and Tables

**Table 1:** Cytokine production in sera of NS1 gene-injected mice with statistically significant difference from vector-injected mice.

Gene	6 h	24 h	48 h	72 h
Abs	TNF-α ↑	ns	IFN-γ ↑	IFN-γ ↑
			IL-10 ↑	
Vas	TNF-α ↑	ns	IFN-γ ↑	IFN-γ ↓
	IL-6 ↑		TNF-α ↑	IL-6 ↓
Sof	IFN-γ ↓	IFN-γ ↑	IFN-γ ↑	ns
	IL-10 ↓		TNF-α ↑	
NS1 *	TNF-α ↑	ns	IFN-γ ↑	IL-10 ↓
			TNF-α ↑	

* Data of all NS1-injected groups were compared to the control (vector injected) group. Non-significant (ns).

## Data Availability

The authors declare that the data generated and analyzed during this study are included in this published article and associated Supplementary Materials. In addition, datasets generated and/or analyzed during the current study are available from the corresponding author on reasonable request.

## Supplementary Materials

The following supporting information can be downloaded at: https://www.mdpi.com/article/10.3390/ijms24021011/s1.
