# Supplementary material for: Activation of Early Proinflammatory Responses by TBEV NS1 Varies between the Strains of Various Subtypes
Source: Int J Mol Sci. 2023 Jan 5;24(2):1011. doi: 10.3390/ijms24021011 (PMC9863113; doi:10.3390/ijms24021011)
Supplement: Supplementary file 1 [file ijms-24-01011-s001.zip › ijms-2120566-supplementary.pdf]

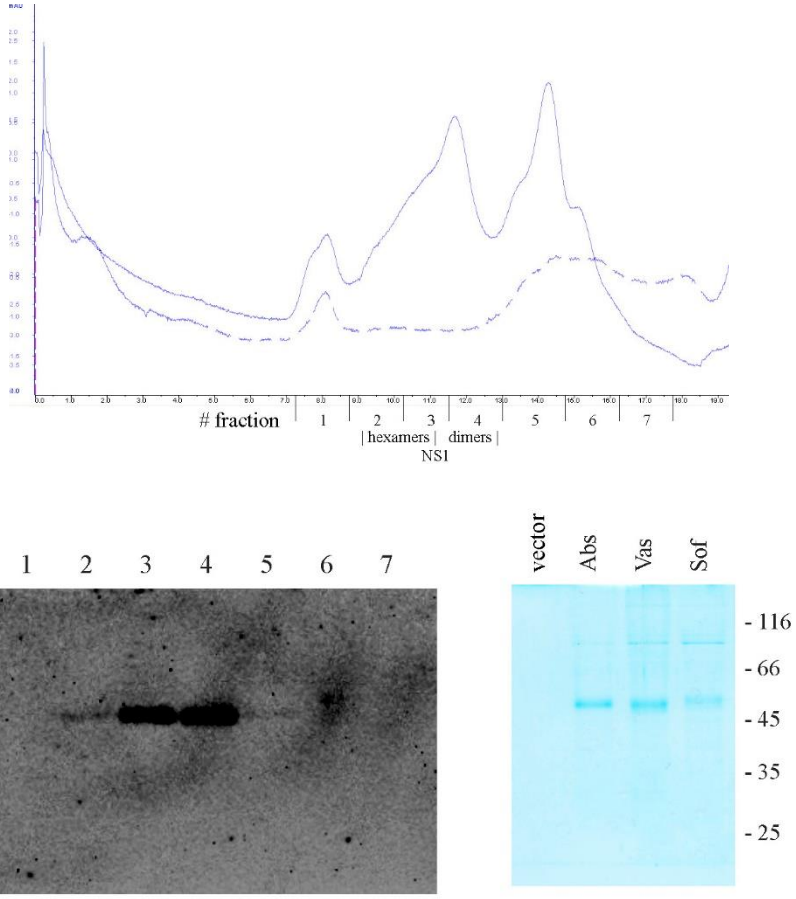

Supplement Figure 1. NS1 protein purification. **(a)** FPLC chromatography on Sephadex 200 of concentrated cell culture medium of NS1 encoding plasmid (solid line) or vector (dotted line) transfected 293T cells. **(b)** Western blot analysis of FPLC fractions with anti-NS1 antibodies (clone 4C4). **(c)** Fraction 3 and 4 were pooled and concentrated. Obtained NS1 samples were resolved by SDS-PAGE and stained with Coomassie blue.
